# Preparation of Cavities for Porcelain

**Published:** 1904-06

**Authors:** W. A. Capon

**Affiliations:** Philadelphia.


### PREPARATION OF CAVITIES FOR PORCELAIN.

By W. A. Capon, Philadelphia.

The preparation of a cavity for porcelain, while similar to that for gold, has one point of difference, which, if not observed, will much lessen the durability of a porcelain filling, and that is the edge. Cut to have the walls perpendicular; avoid saucer-shaped cavities by squaring the bottom and thereby assisting retention. The edges of every cavity or section of tooth against which porcelain is to be placed must have square edges, or, to use the correct term, have the cavity wall as near a right angle to the surface as possible. Of course, in some cases, notably bicuspids and molars, the angle will probably have a tendency to be acute or obtuse, but in all cases have the edges sharp.—*Extract Brief.*

